# On the feasibility of cardiac substructure sparing in magnetic resonance imaging guided stereotactic lung radiotherapy

**DOI:** 10.1002/mp.16028

**Published:** 2022-10-24

**Authors:** Luuk H.G. van der Pol, Sara L. Hackett, Firdaus A.A. Mohamed Hoesein, Louk M.W. Snoeren, Jacqueline Pomp, Bas W. Raaymakers, Joost J.C. Verhoeff, Martin F. Fast

**Affiliations:** ^1^ Department of Radiotherapy University Medical Center Utrecht Heidelberglaan 100, 3584 CX Utrecht The Netherlands; ^2^ Department of Radiology University Medical Center Utrecht Utrecht The Netherlands

**Keywords:** cardiac sparing, cardiac (sub‐)structures, lung SBRT, MR‐linac, treatment planning

## Abstract

**Background:**

Lung stereotactic body radiotherapy (SBRT) has proven an effective treatment for medically inoperable lung tumors, even for (ultra‐)central tumors. Recently, there has been growing interest in radiation‐induced cardiac toxicity in lung radiotherapy. More specifically, dose to cardiac (sub‐)structures (CS) was found to correlate with survival after radiotherapy.

**Purpose:**

Our goal is first, to investigate the percentage of patients who require CS sparing in an magnetic resonance imaging guided lung SBRT workflow, and second, to quantify how successful implementation of cardiac sparing would be.

**Methods:**

The patient cohort consists of 34 patients with stage II–IV lung cancer who were treated with SBRT between 2017 and 2020. A mid‐position computed tomography (CT) image was used to create treatment plans for the 1.5 T Unity MR‐linac (Elekta AB, Stockholm, Sweden) following clinical templates. Under guidance of a cardio‐thoracic radiologist, 11 CS were contoured manually for each patient. Dose constraints for five CS were extracted from the literature. Patients were stratified according to their need for cardiac sparing depending on the CS dose in their non‐CS constrained MR‐linac treatment plans. Cardiac sparing treatment plans (CSPs) were then created and dosimetrically compared with their non‐CS constrained treatment plan counterparts. CSPs complied with the departmental constraints and were considered successful when fulfilling all CS constraints, and partially successful if some CS constraints could be fulfilled. Predictors for the need for and feasibility of cardiac sparing were explored, specifically planning target volume (PTV) size, cranio‐caudal (CC) distance, 3D distance, and in‐field overlap volume histograms (iOVH).

**Results:**

47% of the patients (16 out of 34) were in need of cardiac sparing. A successful CSP could be created for 62.5% (10 out of 16) of these patients. Partially successful CSPs still complied with two to four CS constraints. No significant difference in dose to organs at risk (OARs) or targets was identified between CSPs and the corresponding non‐CS constrained MR‐linac plans. The need for cardiac sparing was found to correlate with distance in the CC direction between target and all of the individual CS (Mann–Whitney *U*‐test *p*‐values <10^−6^). iOVHs revealed that complying with dose constraints for CS is primarily determined by in‐plane distance and secondarily by PTV size.

**Conclusion:**

We demonstrated that CS can be successfully spared in lung SBRT on the MR‐linac for most of this patient cohort, without compromising doses to the tumor or to other OARs. CC distance between the target and CS can be used to predict the need for cardiac sparing. iOVHs, in combination with PTV size, can be used to predict if cardiac sparing will be successful for all constrained CS except the left ventricle.

## 1 INTRODUCTION

Stereotactic body radiotherapy (SBRT) is a recommended alternative for inoperable lung cancer patients.^1^ Peripheral lung tumors have been treated with success, but less success has been achieved for central and ultra‐central lung tumors.^2^ Central lung tumors are defined as tumors within 2 cm of the bronchial tree, and ultra‐central lung tumors are in contact with the bronchial tree.^3^ There are several organs at risk (OARs) in the mediastinum that have to be accounted for during treatment planning and would ideally be visualized at the time of treatment for optimal sparing. The magnetic resonance guided linear accelerator (MR‐linac) facilitates adaptation of the treatment plan to the anatomy at the time of treatment, therefore accounting for any inter‐fractional movement of the tumor relative to the nearby OARs.^4^, ^5^ Treatment using an MR‐linac is expected to be beneficial for SBRT of central and ultra‐central lung tumors, as the proximity of these tumors to radio sensitive structures in the mediastinum can increase the risk of radiation‐induced toxicity.^6^, ^7^

Interest in the radiation‐induced toxicity of the heart in lung cancer was sparked by the results of the Radiation Therapy Oncology Group (RTOG) 0617 trail.^8^ RTOG 0617 was a phase 3 clinical trial investigating dose escalation (74 Gy vs. 60 Gy, both in 2 Gy fractions) for stage III non‐small‐cell lung cancer. Unexpectedly, it was shown that the higher dose arm had lower overall survival (OS). Upon analysis this was linked to dose to the heart, thereby inspiring a quest to better understand radiation‐induced heart disease (RIHD). Various retrospective studies have been conducted investigating RIHD and OS, many of which going beyond whole heart dose metrics, into cardiac (sub‐)structure dose. Different cardiac (sub‐)structures (CS) are assumed to be particularly radio sensitive.^9^, ^10^, ^11^, ^12^, ^13^, ^14^, ^15^, ^16^, ^17^, ^18^, ^19^, ^20^, ^21^ Currently, clinical lung SBRT treatment plans typically constrain the mean heart dose, without considering CS other than the whole heart.

This study investigates whether proposed CS constraints are actionable during treatment planning for an MR‐linac treatment.^4^, ^5^ First, we investigate the fraction of patients with central and ultra‐central lung tumors who exceeded the identified CS constraints and would thus require sparing of CS. Then, we check whether CS constraint implementation would be feasible and what the effect of implementation would be on dose to other structures. Lastly, we explore if the need for and feasibility of CS sparing can be predicted based on patient anatomy.

## 2 METHODS

### 2.1 Patient cohort, pre‐treatment imaging, and contouring

We included 34 patients, with stage II–IV lung cancer, in this retrospective study, who were treated between 2017 and 2020. Further patient characteristics are defined in Table 1. Patients underwent 4D‐computed tomography (CT) imaging (Philips Brilliance Big Bore). The voxelsize is 1 mm by 1 mm by 3 mm in anterior–posterior, left–right, and superior–inferior directions, respectively. All ten respiratory phases were deformably warped into a mid‐position image using in‐house developed software to yield an accurate representation of the time‐weighted anatomy.^22^, ^23^

**TABLE 1:** Characteristics of patient population in this study

Parameter	Value
Age in years, median (range)	71 (31–89)
Male:female, *n* (%)	21:13 (61.7%:38.3%)
Central tumor:ultra central, *n* (%)	16:18 (47.1% :52.9%)
Tumor position in lungs (LSL:LIL:RSL:RML:RIL)	(13:3:9:8:1)
CBCT Linac treatment, *n* (%)	26 (76.5%)
PTV in cm^3^, median (range)	28.1 (1.8–221.5)
GTV in cm^3^, median (range)	9.2 (0.7–147.1)
Tumor stage	II–IV
Year of treatment, range	2017–2020

Abbreviations: CBCT, cone beam computed tomography; GTV, gross tumor volume; LIL, left inferior lobe; LSL, left superior lobe; PTV, planning target volume; RIL, right inferior lobe; RML, right middle lobe; RSL, right superior lobe.

The gross tumor volume (GTV) as well as the conventional OARs (aorta, left and right bronchus, esophagus, heart, lungs, spinal cord, and trachea) were delineated by an experienced radiation oncologist. CS are not routinely included in lung radiotherapy plans, so 11 substructures of interest (ascending aorta, aortic valve, coronary sinus, inferior vena cava, left atrium, left ventricle, pulmonary artery, pulmonary veins, right atrium, right ventricle, and superior vena cava) were manually delineated under supervision of a senior cardio‐thoracic radiologist, following the guidelines of Duane et al.^24^

Twenty‐six of the 34 patients were not treated on the MR‐linac, thus their CT scans were taken with their arms above the head, which requires the manual creation of a region representing the arms to resemble the standard treatment position for the MR‐linac.^25^ The electron density of the artificial arms was set to that of water.

### 2.2 General treatment planning approach

Treatment plans for the Unity MR‐linac (Elekta AB, Stockholm, Sweden) were created using Elekta's Monaco treatment planning system (TPS) (v5.40.01) with a 3 mm resolution dose grid and 3% uncertainty per control point. The TPS uses a Monte Carlo algorithm that is capable of simulating the effects of the 1.5 T magnetic field of the MR‐linac. Treatments were planned as intensity modulated radiotherapy (IMRT), following departmental templates, and consisted of between 8 and 18 co‐planar beams and 27–54 segments, leading to an effective uncertainty per voxel smaller than 0.6%.

Treatment plans created for the MR‐linac avoid beams entering the patient via the arms if possible.^25^ The departmental constraints that were used for treatment planning are given in Supplementary information S12. Patients with central lung tumors received 8 × 7.5 Gy and the ultra‐central lung tumor cases received 12 × 5 Gy, both leading to an accumulated physical dose of 60 Gy to the target. D0.1cc was used in this study as a surrogate for the maximum dose, as the dose distributions calculated with a Monte Carlo algorithm are inherently noisy and the exact maximum dose can fluctuate.

Patient‐specific planning target volume (PTV) margins were calculated following the approach of Ligtenberg et al.^22^ Motion amplitudes for this patient cohort were in the range of 0.06–3.99 mm leading to margins in the range of 3.1–4.7 mm.

### 2.3 Cardiac sparing treatment planning

**FIGURE 1:**
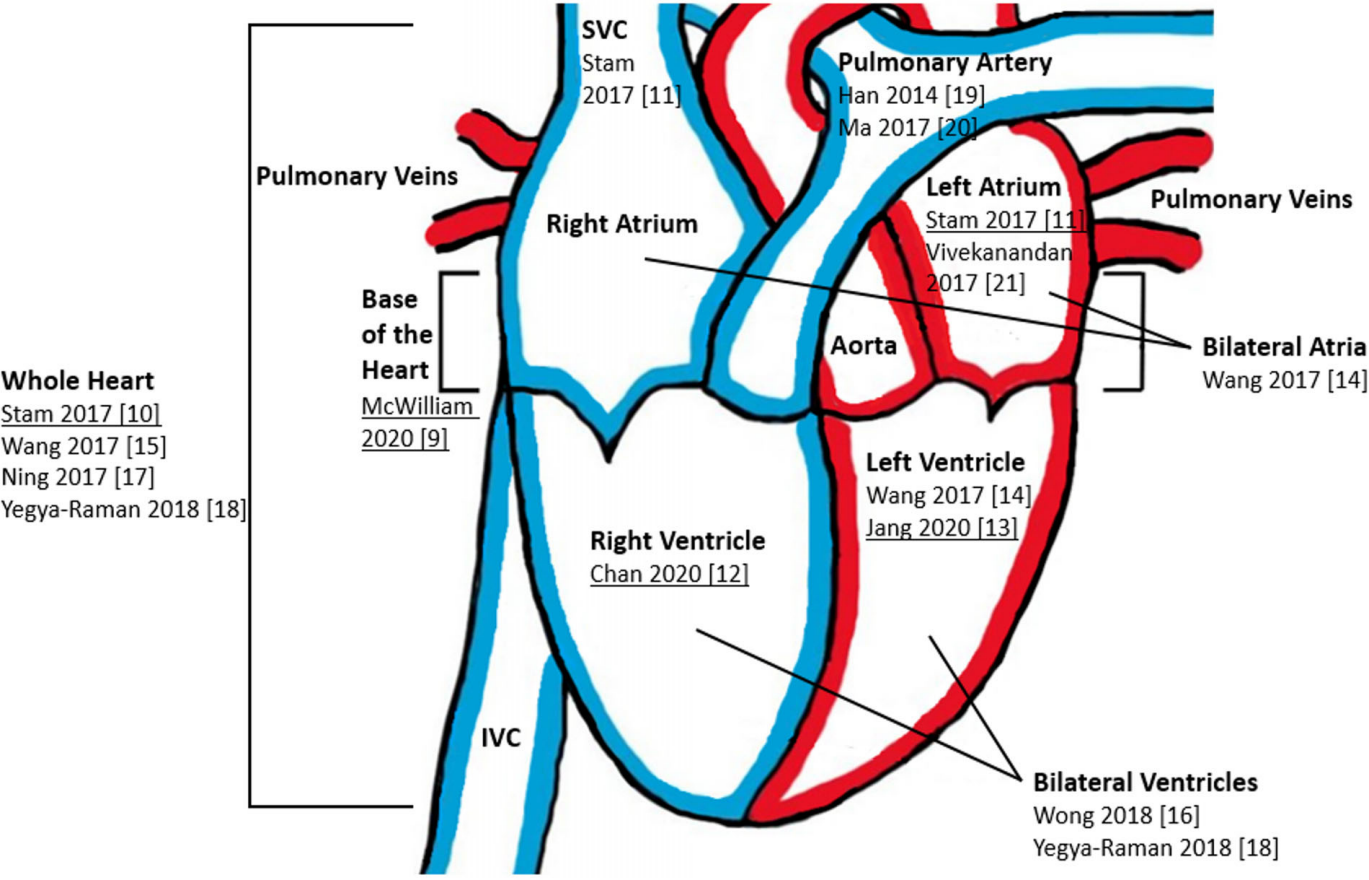
Schematic representation of the heart with CS constraint references displayed. The blue and red color indicate poorly oxygenated and well‐oxygenated blood flow, respectively. The selected constraints are underlined. Abbreviations: IVC, inferior vena cava; SVC, superior vena cava. The base of the heart is defined as the union of the right atrium and ascending aorta. The anatomical definition is given in Supplementary information IX B.

An overview of the radio sensitive CS mentioned in lung radiotherapy studies before 2021 is shown in a schematic representation of the heart in Figure 1. Studies shown in Figure 1 that propose specific constraints were selected for our study. When multiple constraints are proposed for one CS, the most strict constraint was chosen. The constraint on the superior vena cava, by Stam et al.,^11^ was left out as it was considered unfeasibly low at *D*90% < 0.5 Gy. The constraints for the pulmonary artery were not taken into account, as none of the patients were close to exceeding these constraints. The selected CS constraints are shown in Table 2. All patients were stratified according to the identified CS constraints and when a non‐CS constrained treatment plan showed that dose to one or more CS exceeded the constraint, a cardiac sparing plan (CSP) was created. These CSPs were generated by including the CS constraints and modifying beams angles where needed. CSPs were intended to be of similar complexity as non‐CS constrained treatment plans by having a similar amount of beams and the same maximum allowable number of beam segments. An experienced treatment planner reviewed all treatment plans.

**TABLE 2:** Constraints table for cardiac (sub‐)structures (CS)

Author	CS	EQD2 optimal (mandatory) constraint (Gy)
McWilliam 2020^9^	Base of the heart	*D*0.1 cm^3^ < 13.5
Stam 2017^10^	Whole heart	*D*60% < 1.0
Stam 2017^11^	Left atrium	*D*0.1 cm^3^ < 4.7 (6.5)
Chan 2020^12^	Right ventricle	*D*4% < 11 (13.3)
Jang 2020^13^	Left ventricle	*D*0.1 cm^3^ < 60

Any constraints not reported in equivalent dose in 2 Gy fractions (EQD2) were converted using the provided *α*/*β* value for the structure, otherwise a generic *α*/*β* of 2 Gy was used.

### 2.4 Cardiac sparing plan evaluation

CSPs were considered successful when complying with the selected CS constraints (Table 2), and the general departmental planning objectives constraints (Supplementary information S12). Note that for six patients, a clinical decision was made to slightly underdose the target, in order to comply with OAR constraints. In four patient cases with a large target, the clinical decision to slightly overdose the lung‐GTV has been made. Partially successful CSPs also satisfied the departmental constraints, while taking into account the clinical decisions as mentioned, but did not succeed in fulfilling all the CS constraints.

Treatment plans were evaluated based on the dose–volume constraints shown in Table 2 and Supplementary information S12. For each criterion, the dose (converted to equivalent dose in 2 Gy fractions (EQD2)) to the volume specified in the constraint was determined such that one data point per constraint per treatment plan was determined. For analysis constraints were grouped for the CS, conventional OARs and target structures. For patients for whom cardiac sparing was necessary, two DVH points per constraint were determined, one for the original plan and the other for the CSP.

Investigating the differences between the received dose on all structures for the different treatment plans showed the effect of cardiac sparing per individual. Wilcoxon signed‐rank tests were performed to check for significant dose differences between the treatment planning approaches. Bonferoni–Holm correction for multiple testing was applied.

### 2.5 Predictors of cardiac sparing plans

We evaluated PTV size, cranio‐caudal (CC) distance between CS and PTV, 3D distance (shortest distance from the edge of the CS to the PTV) and in‐field overlap volume histograms (iOVHs)^26^, ^27^ as predictor for the need and feasibility of cardiac sparing. iOVHs were created by uniformly expanding of the PTV within the plane of the field and measuring the overlap of the expanded PTV with the CS after each step. The in‐field plane was defined as the volume between planes located 6.4 mm superior and inferior to the edges of the PTV, to account for the dose penumbra in lung.^28^ The stepsize for the iOVH calculation was 1 mm. Receiver operating characteristic (ROC) curves were created for PTV size, CC distance, and 3D distance to investigate how useful a threshold would be in determining the need for and feasibility of cardiac sparing. Significance testing for the relation between PTV size, CC distance, and 3D distance, and the need for and feasibility of cardiac sparing was done using the Mann–Whitney *U*‐test.^29^

## 3 RESULTS

### 3.1 Overview

The non‐CS constrained treatment plans violated the selected CS constraints for 16 of the 34 patients (47%). Of these 16 cases, there were nine with left‐sided tumors and seven with right‐sided tumors. In 10 out of 16 cases (62.5%), the CSP could satisfy all cardiac constraints as well as regular OAR constraints. For the remaining six patients (three with left‐sided tumors and three with right‐sided tumors), the total number of CS constraints that were violated could be reduced from 21 (cumulative over the six non‐CS constrained treatment plans) to 11 (cumulative over the six partially successful cardiac sparing plans). Whenever a CSP was partially successful, the treatment planning decision to sacrifice a certain CS over the other CS had to be made. This decision was based on reaching the maximum number of spared CS.

**FIGURE 2:**
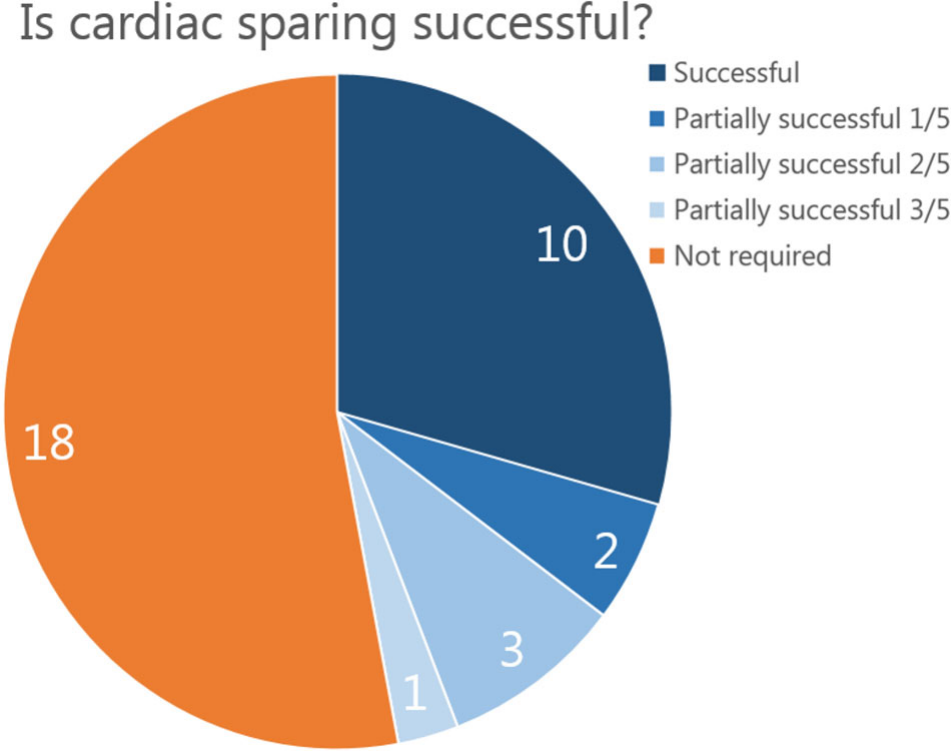
Overview showing the number of patients with successful cardiac sparing. Fractions in the legend indicate the number of CS constraints (out of the total of 5) that were violated.

The CSP for five out of the six patients for whom cardiac sparing was not fully possible, exceeded the constraint for the left atrium, but of these five patients, constraints for other CS were also exceeded in the CSP for four patients. An overview of the patient groups is shown in Figure 2.

The estimated treatment time for CSPs is significantly longer (median increase of 7.3%, Wilcoxon signed‐rank test *p*‐value = 0.04), while there is no significant difference between the number of beams (median decrease of 3.7%, *p*‐value = 0.79), number of segments (identical medians, *p*‐value = 0.72) and number of monitoring units per fraction (median increase of 9.1%, *p*‐value = 0.06).

**FIGURE 3:**
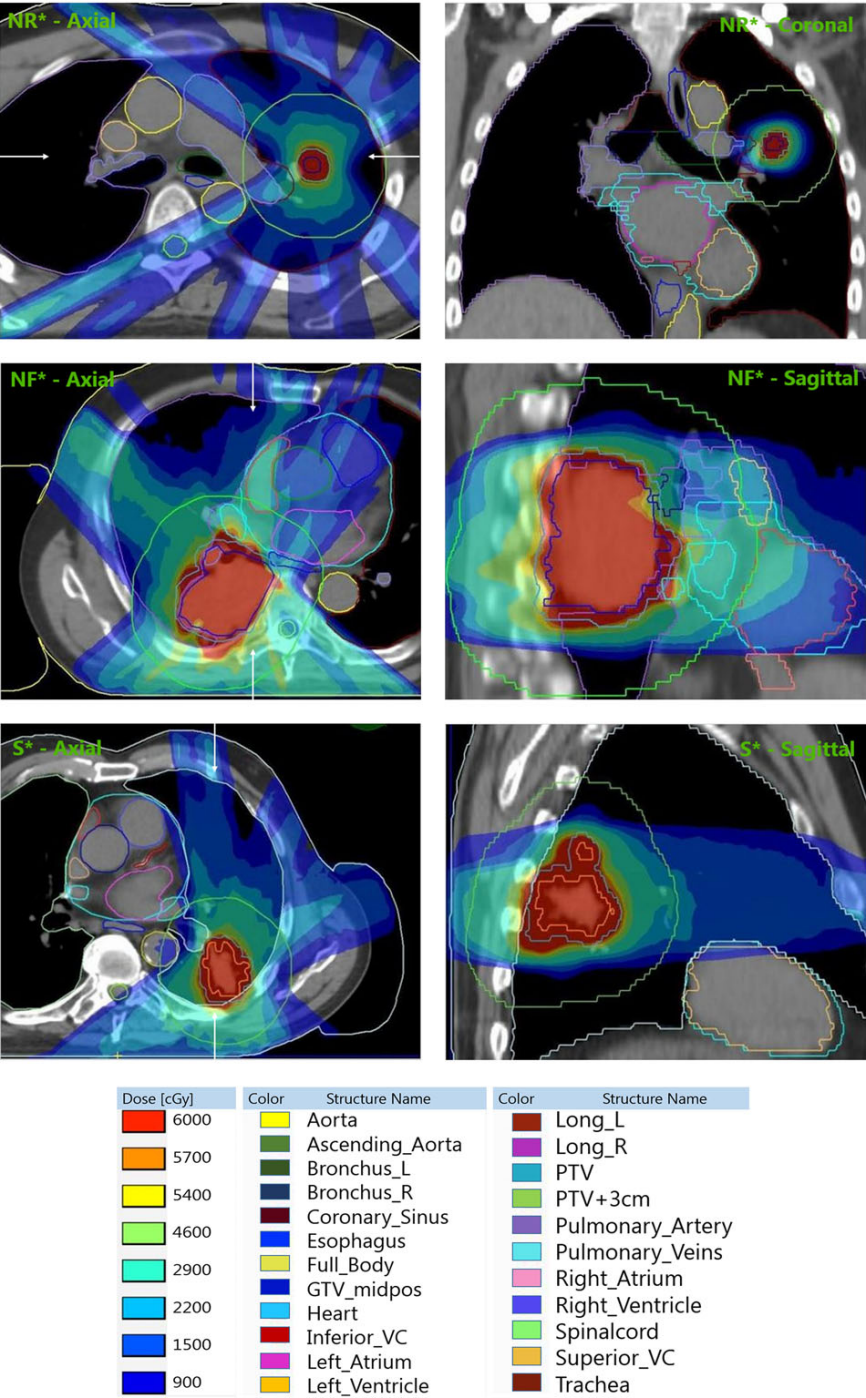
Examples of patient cases for cardiac sparing not required (NR*), not feasible (NF*), and successful (S*), respectively. Computed tomography (CT) scans are shown with a window setting of 500 Hounsfield units (HU) and level of 50 HU.

An example of a patient for whom cardiac sparing is unnecessary because the tumor is located cranially to the CS is shown in the top panel of Figure 3. An example of a patient for whom cardiac sparing was not possible is shown in the middle panel of Figure 3, and the bottom panel of Figure 3 shows an example of a successful cardiac sparing case.

### 3.2 Cardiac sparing plan dose distribution

**FIGURE 4:**
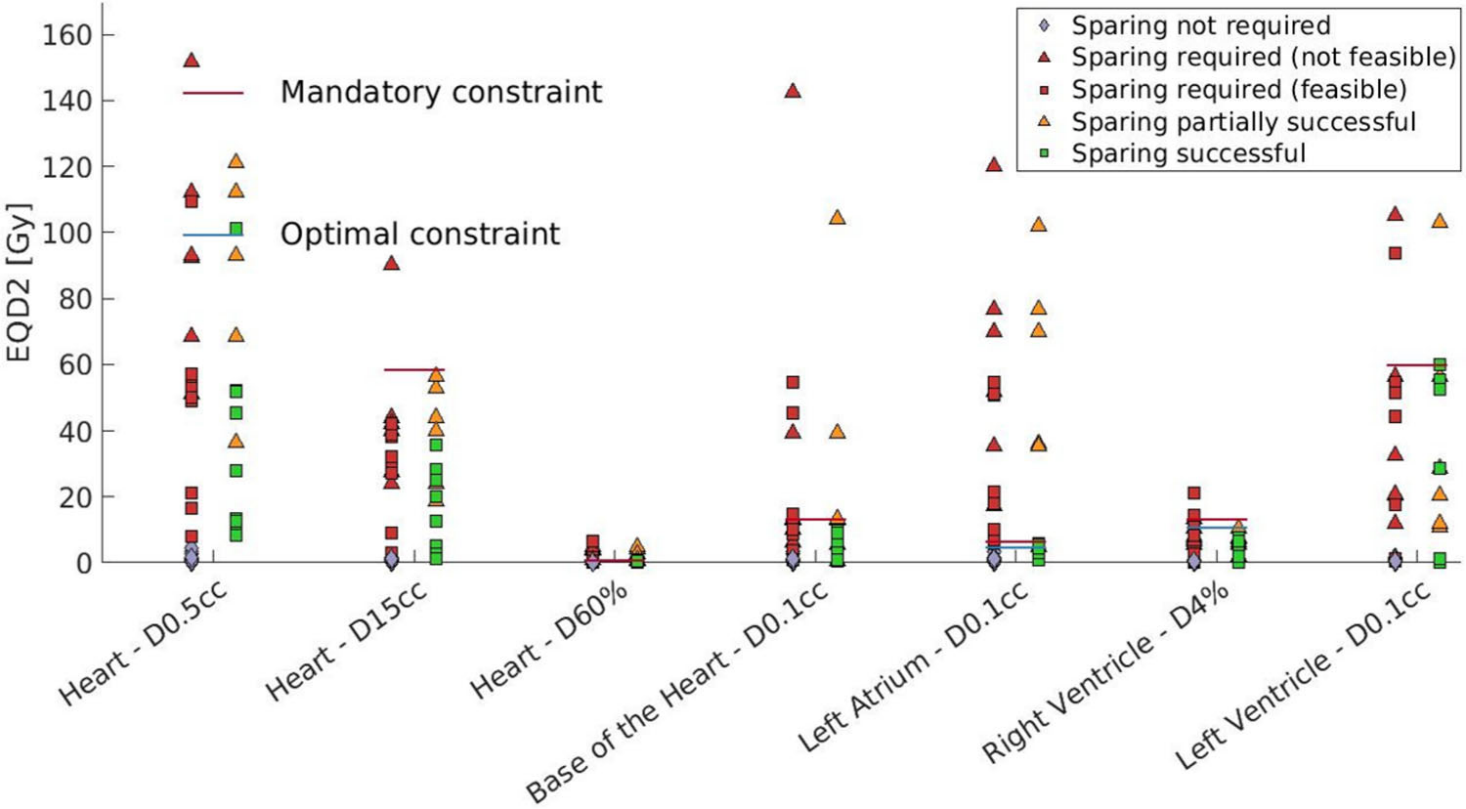
Dose to cardiac (sub‐)structures relative to their constraint level. The left column for each constraint consists of all 34 patients, of which 16 are also present in the right column with their Cardiac sparing treatment plan (CSP). The green and red squares, as well as the red and orange triangles represent different treatment plans for the same patients.

**FIGURE 5:**
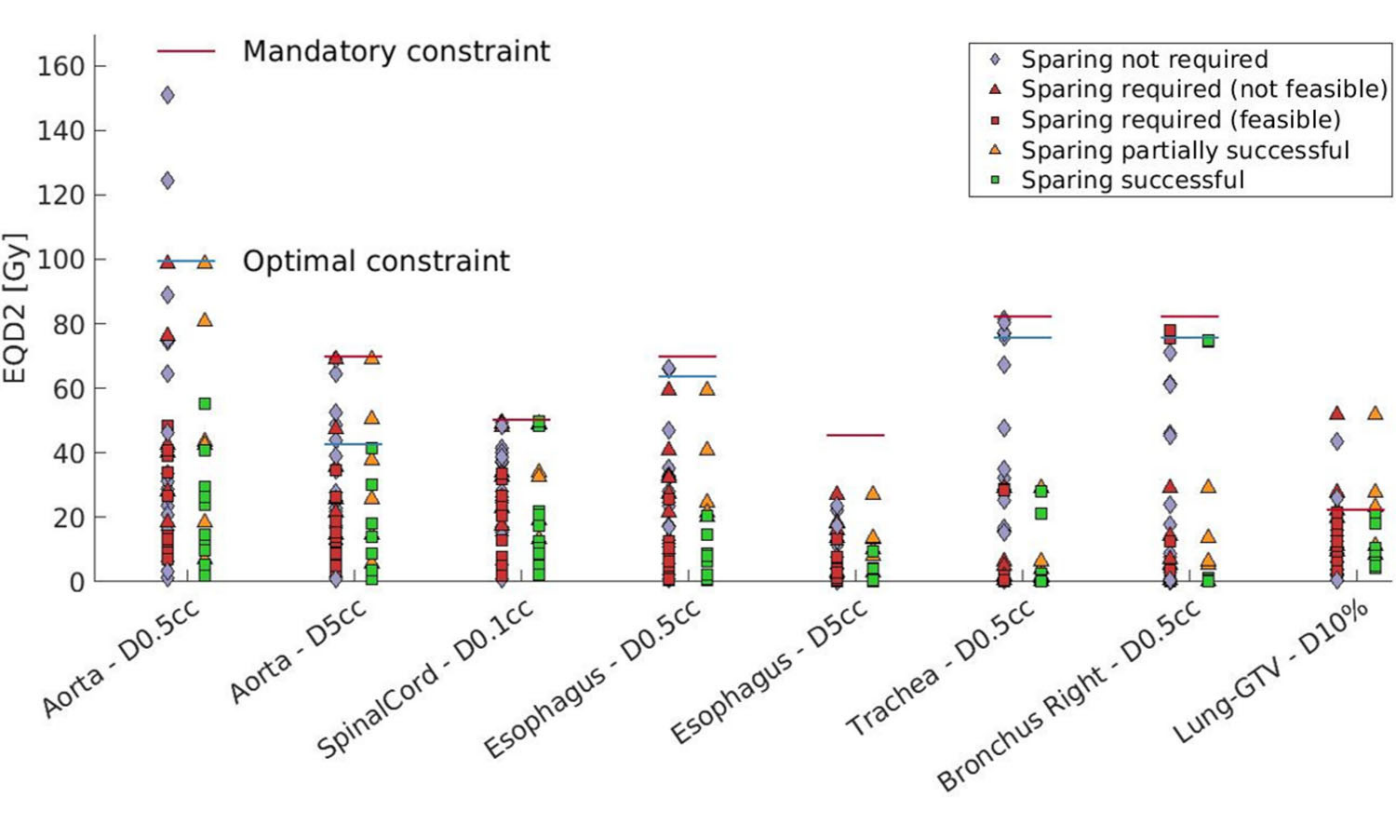
Dose to conventional organs at risk (OARs) relative to their constraint level.

**FIGURE 6:**
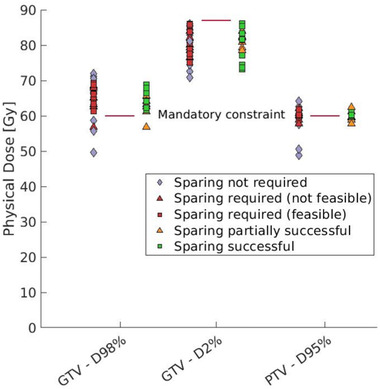
Dose to target structures at their objective (GTV: *D*98% and PTV: *D*95%) or constraint (GTV: *D*2%) level.

Dose to CS, conventional OARs, and target volumes for all non‐CS constrained MR‐linac plans and the CSPs is shown in Figures 4, 5, 6. Figure 4 shows the dose reduction of cardiac sparing for CS, however this is not the case for each patient as highlighted in Supplementary information S2. Figure 5 depicts the effect of cardiac sparing on the conventional OARs, showing the OARs for which it was challenging to meet the constraints. It can be seen that the dose to the conventional OARs in the CSPs is comparable and often lower than the dose to the conventional OARs for the non‐CS constrained MR‐linac plans. No significant differences (Wilcoxon signed‐rank test, alpha = 0.05) in OARs dose were detected between CSP and non‐CS constrained MR‐linac plans after applying the Bonferoni–Holm correction for multiple testing.^30^ Maximum dose to the left atrium and *D*4% to the right ventricle were significantly different when comparing CSPs with non‐CS constrained MR‐linac plans at *p*‐values of 0.01 and 0.04, respectively. Additionally, Figure 6 shows that cardiac sparing does not have a negative effect on the target coverage. Further pair‐wise differences between CSPs and non‐CS constrained MR‐linac plans can be found in Supplementary information IX C.

### 3.3 Predictors for cardiac sparing

All patients who required cardiac sparing had a tumor on the same axial beam entrance plane as the left atrium (Supplementary information IX.5) and exceeded the left atrium constraint in the non‐CS constrained treatment plan. The CC distance between target and all of the constrained CS was significantly lower (Table 3) for the patient who required cardiac sparing with respect to those who did not. However, having a CS in the same axial plane as the target did not result in a clear separation of patients for whom cardiac sparing was achievable. Furthermore, the six patients for whom it was not possible to satisfy all CS constraints, had tumor voxels located within the same axial plane as all of the constrained CS. Nonetheless, five patients with zero CC distance to all of the constrained CS got successful CSPs.

**TABLE 3:** Selection of receiver operator characteristic curve data

PTV size	AUC	*p*‐Value	Threshold (cm^3^)	False positive
Sparing required	0.674	0.088	–	–
Sparing successful (all)	0.863	0.006*	≤ 180	5 (83%)
Sparing successful (required)	0.917	0.005*	≤ 128	3 (50%)
CC distance–sparing required	AUC	*p*‐Value	Threshold (cm^3^)	False positive
Left atrium	1.0	8.74E−08*	= 0	0 (0%)
Base of the heart	0.997	1.44E−07*	≤ 9	2 (11%)
Right ventricle	0.997	3.22E−07*	≤ 27	1 (5.6%)
Left ventricle	0.997	4.41E−07*	≤ 21	1 (5.6%)
Heart	0.998	1.30E−07*	≤ 9	1 (5.6%)
3D distance–sparing successful (required)	AUC	*p*‐Value	Threshold (cm^3^)	False positive
Left atrium	0.933	0.003*	≥ 22	1 (17%)
Base of the heart	0.718	0.181	–	–
Right ventricle	0.750	0.118	–	–
Left ventricle	0.683	0.264	–	–
Heart	0.900	0.008*	≥ 0	6 (100%)
3D distance–sparing successful (all)	AUC	*p*‐Value	Threshold (cm^3^)	False positive
Left atrium	0.941	8.99E−04*	≥ 15	2 (33%)
Base of the heart	0.685	0.168	–	–
Right ventricle	0.780	0.034*	–	–
Left ventricle	0.810	0.020*	≥ 1	5 (83%)
Heart	0.958	5.48E−04*	≥ 0	6 (100%)

(All) and (required) mean all patients and patients that require sparing, respectively. Abbreviations: AUC, area under curve; PTV, planning target volume. Symbol * means significant *p*‐value (≤ 0.05) as a result of Mann–Whitney *U*‐test. Thresholds are only determined when AUC ≥ 0.8^37^ and *p*‐value is significant. False positive shows the number (percentage) of patients that would be classified incorrectly based on the displayed threshold.

The median PTV size of the patients for whom cardiac sparing was not fully achievable is 122 cm^3^ (range: 29–222 cm^3^), while the group of patients for whom cardiac sparing was possible has a median PTV size of 24 cm^3^ (range: 10–127 cm^3^). The patient with a 127 cm^3^ target for whom cardiac sparing was fully achievable, has a distance of more than 5 cm between the edge of the PTV and all CS.

**FIGURE 7:**
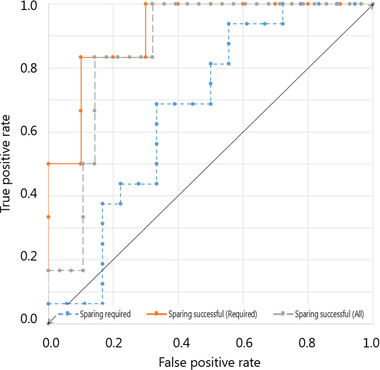
Receiver operating characteristic (ROC) curves for PTV size. The diagonal (double arrow headed) line indicates a random classifier.

ROC curves for decisions based on PTV size are shown in Figure 7, while ROC curves for 3D distance and CC distance can be found in Supplementary information IX E and IX F, respectively. The data from the ROC curves with the highest area under the curve (AUC) and a false positive rate that is not 1 for all CS, is displayed in Table 3. The complete table can be found in Supplementary information S13. Thresholds were determined at a true positive rate (TPR) of 1, to ensure that the maximum achievable sparing will be reached. The cost of ensuring maximum achievable sparing would be the unnecessary exploration of CSPs for patients not in need of cardiac sparing and trying to achieve complete sparing for patients who cannot get it. This cost is displayed in Table 3 in the false positive column.

**FIGURE 8:**
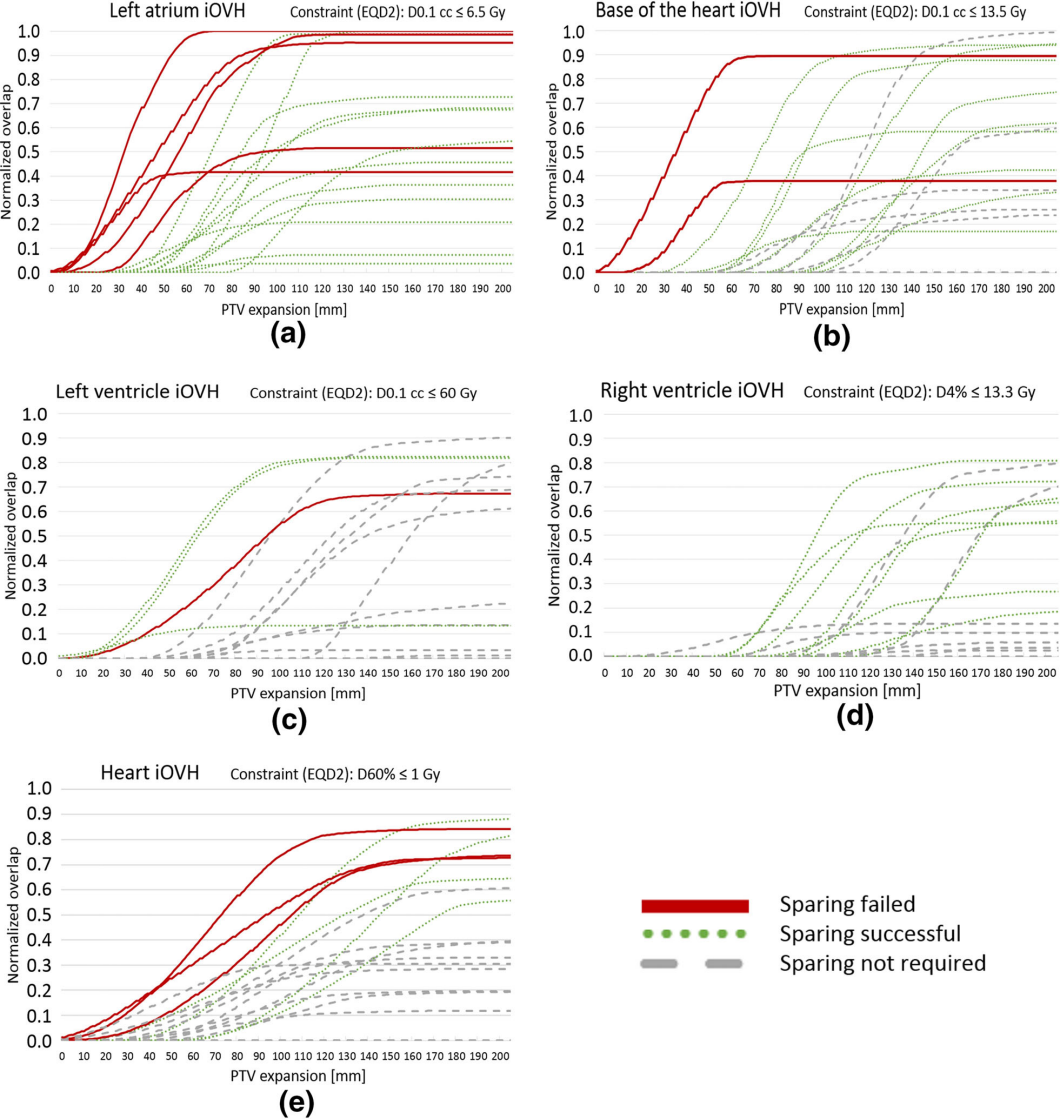
In‐field overlap volume histograms (iOVHs) for the left atrium (a), base of the heart (b), left ventricle (c), right ventricle (d), and heart (e), including all patients that require sparing for one or more CS

iOVHs show that the need and ability to spare the left atrium is dependent on the minimum distance to the target, as is expected from a maximum dose constraint (Figure 8(a)). For the base of the heart only the feasibility of sparing depends on the minimum distance to the target, whereas the need for sparing is more ambiguous (Figure 8(b)). The need for sparing of the left ventricle is dependent on the minimum distance to the target, as expected with a maximum dose constraint (Figure 8(c)). However, the ability to spare the left ventricle cannot be explained based on the iOVH and PTV size. The iOVH for the right ventricle (Figure 8(d)) shows why all patients could get sparing. Only one patient had their target within 5 cm of the right ventricle, but that patient had little overlap.

The relationship between overlap volume and the need and ability to spare the whole heart is less apparent. Figure 8(e) shows that closest distance was not decisive. Patients with smaller total in‐field overlap (0.4 or lower) with the heart do not need sparing but higher amount of total in‐field overlap is mixed between successful and failed sparing. The median PTV size for those patients who fail the additional heart constraint and those who succeed with the additional heart constraint are 99 and 23 cm^3^, respectively.

## 4 DISCUSSION

To our knowledge, this study is the first to investigate the need for and feasibility of cardiac sparing lung SBRT on the unity MR‐linac. The exploration of treatment plans for 34 patients showed that just under half of the patients (47%) are in need of cardiac sparing. From the patients in need of cardiac sparing, more than half (62.5%) successfully reach cardiac sparing by designing new treatment plans. The downside of the new treatment plans is the slightly longer (7.3%) delivery time. The patients for whom a CSP is not fully achievable have, on average, much larger PTVs.

The CS constraints used in this study were selected from a range of sometimes contradictory publications, of which the majority has OS as endpoint rather than cardiac events. Therefore, the CS constraints used are subject to future revision. A recent review by Zhang et al.^31^ shows that over 90 unique cardiac dosimetric parameters have been investigated, highlighting that uniform guidelines are yet to be determined. Furthermore, during the conduction of this study additional CS constraints have been published, indicating that more clinical evidence is emerging.^32^, ^33^, ^34^ As earlier research indicates, radio‐sensitivity differs between CS and therefore specific CS sparing would be beneficial over simply reducing the mean heart dose. Therefore, the goal of this study was to determine if the selected CS constraints can be met on the MR‐linac by adapting the treatment planning approach. Major changes in the CS constraints could mean a shift in the trade‐off that was made, rendering current CSPs suboptimal and requiring the cardiac sparing treatment planning approach to be revisited.

Many of the proposed CS constraints are (near) maximum dose constraints, necessitating precise daily visualization of the anatomy as small shifts of CS can cause large differences in dose. An MR‐linac treatment would solve this difficulty, whereas conventional cone beam CT could not visualize all of the CS. Many of the included patients were not treated on the MR‐linac, as the clinical MR‐linac lung SBRT treatment was still in workup during the majority of patient inclusion. However, the retrospective inclusion led to the selection of patients with (ultra‐)central tumors, who would be considered for MR‐linac treatment in today's clinic.

Naively, protecting parts of the heart from dose could result in higher (still below constraint) dose to surrounding OARs, as maintaining target coverage is the first priority during plan optimisation. However, our results show that conventional OAR dose and target coverage are not penalized in cases where CS sparing is possible (Figures 5 and 6). Only the lung‐GTV structure typically receives a lower dose in a non‐CS constrained MR‐linac plan. This might be explained by the location of the heart, as it lies in close proximity to many of the mediastinal OARs. Therefore, avoiding the heart will also avoid the other OARs, but additional dose might be deposited in the ipsilateral lung. The magnitude of dose redistribution depends on the tumor location. Further analysis (displayed in Supplementary information S4) reveals that there is not a single patient for whom the dose to all conventional OARs was reduced in the CSP. Therefore, it is clear that CSPs necessitate a trade‐off between doses to conventional OARs. )

A trade‐off between doses to CS also takes place; a dose difference graph (Supplementary information S2) revealed that, for individual patients, a few CS received more dose in the CSP compared to the non‐CS constrained MR‐linac plan. Presumably, the dose to the CS that need sparing the most is decreased at the cost of additional dose to other CS. Some patients did not reach the desired target coverage in their non‐CS constrained MR‐linac treatment plans due to constraints on conventional OARs, which we prioritized. As the whole heart is included in the conventional OARs, CSPs only expand on sparing the heart and are therefore considered fairly safe. Differences in target coverage between the treatment planning approaches are minimal, indicating that cardiac sparing does not necessarily have a detrimental effect on target coverage.

Predicting the need for and feasibility of cardiac sparing based on the anatomy alone would be beneficial as creating treatment plans and exploring the need and feasibility of cardiac sparing is a time‐consuming task, which could be cumbersome in an online adaptive treatment setup such as with the MR‐linac.

Several simple predictors were investigated because of the following reasons: PTV size could be predictive for the need for and feasibility of cardiac sparing, as larger treatment target have a broader dose penumbra.^35^, ^36^ PTV size alone did not explain the entire cardiac sparing needs and capabilities. Distance metrics could be required to complete the predictions. 3D distance is an intuitive choice, as a treatment target close to OARs will lead to difficulty in sparing those OARs which is no different for CS. The anticipated predictive effect of CC distance is based on the fact that treatment is delivered by co‐planar beams. Therefore, a CS being out of the treatment plane will receive little to no dose.

CC distance is shown to predict the need for cardiac sparing perfectly (Table 3) but cannot predict the success of cardiac sparing, as shown in the results and revealed by the ROC curves (Supplementary information IX F and S13). Determining that cardiac sparing is needed is essential, as creating CSPs for patients for whom it is not necessary is inefficient. Thresholds in ROC curves were only determined when *p*‐values were significant (*p* <0.05) and AUC was above 0.8.^37^ Thresholds were chosen at a TPR of 1, which ensures that only the cases which do not need the extra effort (either not in need for or not succeeding in cardiac sparing), are added to the respective group. This will result in the best CSP for every patient at the cost of additional exploration of treatment plans for patients that turn out not to need or succeed at cardiac sparing. The cost that would arise from the patient cohort in this study can be seen in the false positive column of Table 3. The time penalty would be more important in the practical MR‐linac workflow, which could result in a different threshold.

Interestingly, PTV size alone, independent of the location with respect to the heart, is already indicative of CSP success, however it cannot predict if sparing is required. Likewise, 3D distance is unable to determine if cardiac sparing is needed but can predict successful and partially successful CSPs (Table 3), even more accurately compared to PTV size. Combining the predictions for the need of sparing by CC distance with the ability to succeed in sparing by 3D would give the most accurate prediction. However, 3D distance correlates with CC distance which influences the prediction, while lacking the predictive value of PTV size. These shortcomings are solved with the use of iOVHs. Besides showing the minimum in‐plane distance between the target and a structure of interest, iOVHs reveal the percentage of a structure that is in the same axial plane as the target and therefore at risk of getting substantial dose. iOVHs displayed in this study (Figure 8) could be used as a reference for future cases of cardiac sparing with similar CS constraints. When the iOVHs of two structures with comparable (but not equal) constraints with similar distance distributions would be compared, violation of the stricter constraints will occur at distances farther away from the structure of interest. The main predictor of the ability to spare the left atrium and the base of the heart is minimum distance. To predict the feasibility of the additional whole heart constraint, the knowledge of the total in field overlap that is reached is beneficial but the PTV sizes are necessary to further stratify into sparing successful and sparing failed. OVH selection based on PTV size has been shown to be useful in the past.^38^ All of the patients were able to reach sparing for the right ventricle, mainly because of the distance to the PTV. Having more distance to the right ventricle in comparison with the other constrained CS is a characteristic of this patient group, as they all had central or ultra‐central tumors. These tumors are in close proximity to the bronchial tree, from which the right ventricle is the most distant of the four chambers. This was also reflected in the tumor positions as given in Table 1.

The need for sparing of the left ventricle is clearly based on distance, however, more information is required to explain how sparing succeeds for cases that have higher in‐field overlap at a similar distance, as seen in the left ventricle iOVH. Of the two successful sparing cases with lines above the sparing failed case in the left ventricle iOVH (Figure 8(c)), one case is explained by a difference of a factor 2 in PTV size, while also having a distance that is 5 mm more compared to the failed case.

When comparing the CSP of the patient for whom sparing was not possible for the left ventricle (referred to as patient 1) with the CSP of the other patient represented with a higher‐level line in the iOVH (referred to as patient 2), the unexpected difference in ability to spare the left ventricle can be explained. Patient 1 only fails the constraint for the left ventricle, where patient 2 fails two other constraints. Looking into the CSP shows that patient 1 meets all other CS constraints, while in the CSP for patient 2, two other CS constraints fail in any case. This allows the CSP for patient 2 to have more dose put into CS for which the constraint was already failed, in order to save the left ventricle. In this specific comparison, the left atrium D0.1cc for patient 2 is more than 10 times higher than the left atrium D0.1cc for patient 1. Additionally, target coverage for patient 2 was slightly below the standard requirement at 98% and 93% for GTV $V_{60\;\text{Gy}}$ and PTV $V_{60\;\text{Gy}}$, respectively. This additional explanation required for the iOVH for the left ventricle highlights the limitation of the iOVH approach as a predictor.

CSPs have been made before by Morris et al.^39^ However, their goal was different from ours, as they aimed to reduce dose to the CS receiving the highest dose in the original plan with the overall objective to minimize substructure dose, in contrast to our approach in which specific cardiac constraints were selected and enforced during treatment planning. Furthermore, they actively managed and investigated differences in plan complexity while we keep this within the same limits as the non‐CS constrained treatment plans and thereby only slightly increased treatment time.

An important consideration for future research is the effect of cardiorespiratory motion on the dose distribution and how it may affect cardiac sparing. The motion of CS has been investigated recently by Morris et al.^40^ For the chambers, median displacements were 1.8, 1.9, and 2.2 mm in the left–right, anterior–posterior, and superior–inferior axis, respectively. This leads to planning organ at risk volumes (PRVs) with 3–5 mm anisotropic substructure‐specific margins. Furthermore, the effect of respiratory motion on dose to the heart and CS has been explored by Miller et al.^41^ (The maximum vector displacements ranged from 5 to 10 mm across most substructures), however this was not in lung cancer patients alone. Motion effects can be mitigated by using PRVs (similar to Morris et al.), using breath‐hold (at risk of larger displacement when lack of compliance occurs^40^) or by using active motion management.^42^

## 5 CONCLUSION

We demonstrated that cardiac (sub‐)structures can be successfully spared in lung SBRT on the MR‐linac for the majority of this patient cohort (82%, 28 out of 34 cases), without compromising doses to the tumor or to other OARs. The need for cardiac sparing can be perfectly predicted using the CC distance between the PTV and the CS. Furthermore, iOVHs can be used to predict the feasibility of sparing of individual CS and are largely based on the in‐plane distance, while being influenced by PTV size. CS constraints regarding large volumes are more dependent on PTV size and total in‐field overlap.

## CONFLICT OF INTEREST

The authors declare that there is no conflict of interest that could be perceived as prejudicing the impartiality of the research reported.

## Supporting information

Supporting Information

Supporting Information

Supporting Information

Supporting Information

Supporting Information

Supporting Information

Supporting Information

Supporting Information

Supporting Information

Supporting Information

Supporting Information

Supporting Information

Supporting Information
