# Supplementary material for: On the feasibility of cardiac substructure sparing in magnetic resonance imaging guided stereotactic lung radiotherapy
Source: Med Phys. 2022 Oct 24;50(1):397–409. doi: 10.1002/mp.16028 (PMC10092491; doi:10.1002/mp.16028)
Supplement: Supplementary file 12 — Supporting Information [file MP-50-397-s011.doc]

Table IV: Constraints table for conventional OARs and target volumes.

| **Organ at risk** | **EQD2 optimal (mandatory) constraint [Gy]** |
| --- | --- |
| Aorta | D0.5cc < 49 (65) |
| Aorta | D5cc < 30 (40) |
| Spinal Cord | D0.1cc < 33 |
| Esophagus | D0.5cc < 38 (40) |
| Esophagus | D5cc < 31 |
| Trachea | D0.5cc < 42 (44) |
| Bronchus (Left) | D0.5cc < 42 (44) |
| Bronchus (Right) | D0.5cc < 42 (44) |
| Heart | D0.5cc < 49 (60) |
| Heart | D15cc < 37 |
| Plexus | D0.1cc < 36 (37) |
| Skin | D0.5cc < 49 |
| Skin | D10cc < 44 |
| **Target volume** | **Physical dose constraint [Gy]** |
| GTV | D99.9% > 60 |
| PTV | D95% > 60 |

Constraints were converted to EQD2 using an α/β of 2 Gy. Different α/β are reported in literature but for consistency the value of 2 Gy was chosen \cite{MCWILLIAM20201073, SCHEENSTRA2014224, FLEMING2016423}.
