# Supplementary material for: On the feasibility of cardiac substructure sparing in magnetic resonance imaging guided stereotactic lung radiotherapy
Source: Med Phys. 2022 Oct 24;50(1):397–409. doi: 10.1002/mp.16028 (PMC10092491; doi:10.1002/mp.16028)
Supplement: Supplementary file 13 — Supporting Information [file MP-50-397-s008.doc]

Table V: Complete receiver operator characteristic curve data.

| **PTV size** | AUC | p-value | Threshold [cc] | False positive |
| --- | --- | --- | --- | --- |
| Sparing required | 0.674 | 0.088 | - | - |
| Sparing successful (All) | 0.863 | 0.006* | ≤ 180 | 5 (83%) |
| Sparing successful (Required) | 0.917 | 0.005* | ≤ 128 | 3 (50%) |
| **CC distance – Sparing required** | AUC | p-value | Threshold [mm] | False positive |
| Left atrium | 1.0 | 8.74E-08* | = 0 | 0 (0%) |
| Base of the heart | 0.997 | 1.44E-07* | ≤ 9 | 2 (11%) |
| Right ventricle | 0.997 | 3.22E-07* | ≤ 27 | 1 (5.6%) |
| Left ventricle | 0.997 | 4.41E-07* | ≤ 21 | 1 (5.6%) |
| Heart | 0.998 | 1.30E-07* | ≤ 9 | 1 (5.6%) |
| **CC distance - Sparing successful (Required)** | AUC | p-value | Threshold [mm] | False positive |
| Left atrium | 0,5 | 1 | - | - |
| Base of the heart | 0,5455 | 1 | - | - |
| Right ventricle | 0,7273 | 0.149 | - | - |
| Left ventricle | 0,7273 | 0.149 | - | - |
| Heart | 0,5455 | 1 | - | - |
| **CC distance - Sparing successful (All)** | AUC | p-value | Threshold [mm] | False positive |
| Left atrium | 0,8214 | 0,011* | ≥ 0 | 6 (100%) |
| Base of the heart | 0,8393 | 0,008* | ≥ 0 | 6 (100%) |
| Right ventricle | 0,9107 | 0,002* | ≥ 0 | 6 (100%) |
| Left ventricle | 0,8929 | 0,003* | ≥ 0 | 6 (100%) |
| Heart | 0,8214 | 0,011* | ≥ 0 | 6 (100%) |

Table V Continued:

| **3D distance - Sparing required** | AUC | p-value | Threshold [mm] | False classification |
| --- | --- | --- | --- | --- |
| Left atrium | 0,75 | 0,014* | - | - |
| Base of the heart | 0,5313 | 0,769 | - | - |
| Right ventricle | 0,6979 | 0,051 | - | - |
| Left ventricle | 0,7917 | 0,004* | - | - |
| Heart | 0,8264 | 0,001* | ≤ 54 | 12 (67%) |
| **3D distance - Sparing successful (Required)** | AUC | p-value | Threshold [mm] | False classification |
| Left atrium | 0,9333 | 0,003* | ≥ 22 | 1 (17%) |
| Base of the heart | 0,7176 | 0,181 | - | - |
| Right ventricle | 0,75 | 0,118 | - | - |
| Left ventricle | 0,6833 | 0,264 | - | - |
| Heart | 0,9 | 0,008* | ≥ 0 | 6 (100%) |
| **3D distance - Sparing successful (All)** | AUC | p-value | Threshold [mm] | False classification |
| Left atrium | 0,9405 | 8,99E-04* | ≥ 15 | 2 (33%) |
| Base of the heart | 0,6845 | 0,168 | - | - |
| Right ventricle | 0,7798 | 0,034* | - | - |
| Left ventricle | 0,8095 | 0,020* | ≥ 1 | 5 (83%) |
| Heart | 0,9583 | 5,48E-04* | ≥ 0 | 6 (100%) |

(All) and (Required) mean all patients and patients that require sparing respectively. AUC = area under curve. * means significant p-value (≤ 0.05) as a result of Mann-Whitney U test. Thresholds are only determined when AUC ≥ 0.8 \cite{ROCrequiredAUC08} and p-value is significant. False positive shows the number (percentage) of patients that would be classified incorrectly based on the displayed threshold.
